# Screening and management of pre-eclampsia and eclampsia in antenatal and labor and delivery services: findings from cross-sectional observation studies in six sub-Saharan African countries

**DOI:** 10.1186/s12884-018-1972-1

**Published:** 2018-08-23

**Authors:** Barbara Rawlins, Marya Plotkin, Jean Pierre Rakotovao, Ashebir Getachew, Maria Vaz, Jim Ricca, Pam Lynam, Frank Kagema, Patricia Gomez

**Affiliations:** 1 Jhpiego, 1615 Thames Street, Baltimore, MD 21231 USA; 2 Jhpiego Madagascar, Antananarivo, Madagascar; 3 Gandhi Memorial Hospital, Addis Ababa, Ethiopia; 4 Jhpiego Mozambique, Maputo, Mozambique; 5 Kenyatta Referral and Teaching Hospital, Nairobi, Kenya

**Keywords:** Pre-eclampsia, Eclampsia, Quality of care, Antenatal care, Labor and delivery, Ethiopia, Kenya, Tanzania, Rwanda, Mozambique, Zanzibar

## Abstract

**Background:**

Preeclampsia and eclampsia (PE/E) are major contributors to maternal and neonatal deaths in developing countries, associated with 10–15% of direct maternal deaths and nearly a quarter of stillbirths and newborn deaths, many of which are preventable with improved care. We present results related to WHO-recommended interventions for screening and management of PE/E during antenatal care (ANC) and labor and delivery (L & D) from a study conducted in six sub-Saharan African countries.

**Methods:**

From 2010 to 2012, cross-sectional studies which directly observed provision of ANC and L & D services in six sub-Saharan African countries were conducted. Results from 643 health facilities of different levels in Ethiopia (*n* = 19), Kenya (*n* = 509), Madagascar (*n* = 36), Mozambique (*n* = 46), Rwanda (*n* = 72), and Tanzania (*n* = 52), were combined for this analysis. While studies were sampled separately in each country, all used standardized observation checklists and inventory assessment tools.

**Results:**

2920 women receiving ANC and 2689 women in L & D were observed. Thirty-nine percent of ANC clients were asked about PE/E danger signs, and 68% had their blood pressure (BP) taken correctly (range 48–96%). Roughly half (46%) underwent testing for proteinuria. Twenty-three percent of women in L & D were asked about PE/E danger signs (range 11–34%); 77% had their BP checked upon admission (range 59–85%); and 6% had testing for proteinuria. Twenty-five cases of severe PE/E were observed: magnesium sulfate (MgSO4) was used in 15, not used in 5, and for 5 use was unknown. The availability of MgSO4 in L & D varied from 16% in Ethiopia to 100% in Mozambique.

**Conclusions:**

Observed ANC consultations and L & D cases showed low use of WHO-recommended practices for PE/E screening and management. Availability of MgSO4 was low in multiple countries, though it was on the essential drug list of all surveyed countries. Country programs are encouraged to address gaps in screening and management of PE/E in ANC and L & D to contribute to lower maternal and perinatal mortality.

**Electronic supplementary material:**

The online version of this article (10.1186/s12884-018-1972-1) contains supplementary material, which is available to authorized users.

## Background

Pre-eclampsia/eclampsia (PE/E) is one of the leading causes of maternal, fetal and neonatal morbidity and mortality. It affects 2–8% of pregnancies worldwide, is associated with 10–15% of direct maternal deaths and up to 25% of stillbirths and newborn deaths in developing countries [1]. A systematic review covering 40 countries and 39 million women found an incidence of 4.6% of all deliveries for preeclampsia and 1.4% for eclampsia, while a secondary analysis of WHO data found the combined PE/E prevalence to be 4% [2]. There was high regional variation, with sub-Saharan Africa having the highest PE/E prevalence at 5.6% and a crude incidence of 2.9% [3].

Although the cause of PE/E are not well understood, the majority of cases will resolve upon delivery of the fetus and placenta. PE/E can also occur postpartum, with up to 26% of eclamptic seizures occurring beyond 48 h and as late as six weeks following delivery [4]. Screening for pre-eclampsia, which should occur during ANC, upon admission to and throughout L & D, includes measurement of blood pressure and urine protein, and enquiring about PE/E related danger signs [5].

Improving quality of maternal and newborn health care is a central focus of efforts to achieve Sustainable Development Goals (SDG) for maternal and newborn health [6]. In 2015 an estimated 303,000 women died in childbirth or from pregnancy related causes such as PE/E, and most of these deaths are preventable with high quality maternal and newborn services [7]. PE/E is no exception. Over 50,000 maternal deaths per year are associated with PE/E [1], and improving the quality and availability of PE/E screening and management in maternal and newborn care services could save many of these lives.

Quality of maternal and newborn health care is often not well documented or understood. As described in WHO’s 2014 consultation on quality of maternal and newborn care: “… there has been limited progress in improving maternal and pediatric outcomes because of a major gap between coverage and the quality of care provided in health facilities” [8]. Some countries with high coverage of skilled attendance at birth still have high levels of maternal mortality [9], indicating that while more women are attended by a skilled health worker, quality of care (QoC) may be poor. Assessing quality of maternal and newborn care is a global priority, and in a 2013 consultation experts defined global indicators to assess QoC in maternity, newborn and child health services [8], and these have been further refined and described in WHO’s Core 100 indicators [10].

Clinical management of severe pre-eclampsia and eclampsia during pregnancy, labor and delivery involves administration of parenteral magnesium sulfate (MgSO4) for prevention or management of convulsions and anti-hypertensive medications. The other key component is timing and route of delivery based on factors such as gestational age.

WHO recommends MgSO4 for management of PE/E [5]. The scientific basis for the recommendation came from evidence generated through the Magnesium Sulfate for Prevention of Eclampsia (Magpie) trial, which compared MgSO4 to placebo for treatment of PE/E. The multi-country study showed that MgSO4 halved the risk of recurrent eclampsia, and probably reduced the risk of maternal death, with no substantive harmful effects to mother or baby [11].

Both quality and level of care can make a difference in management of PE/E. Lower level health facilities in resource-limited settings may provide the same screening for PE/E during antenatal and delivery services as higher level facilities, but may refer women who are found to have PE/E. Some services, such as a surgical theatre to perform a caesarean section, or a laboratory to conduct urine testing for protein, may be unavailable at lower level health facilities. In addition to infrastructure, lower level facilities may have different management protocols as designated by the Ministry of Health.

Quality improvement efforts related to screening for PE/E in ANC and management of PE/E in labor and delivery services have suffered from a lack of understanding of actual practices in the health facility setting in sub-Saharan Africa. There are a few standardized assessments of the quality of maternal and newborn health care that have been applied in multiple countries. These include the Averting Maternal Death and Disability (AMDD) studies [12], the Service Availability and Readiness Assessments (SARA) [13], and Measure DHS Service Assessments [14]. These surveys, which primarily or solely focus on facility readiness to provide maternal and newborn care, are largely conducted using facility audits of human resources, availability of equipment and commodities, and self-reported practices of health workers, but contain no observational component.

To address this information gap, from 2009 to 2015 the USAID-funded Maternal and Child Health Integrated Program (MCHIP) conducted cross-sectional quality of care surveys, using direct observation of antenatal care consultations and labors and deliveries. The QoC surveys were unique in their utilization of direct observation to assess the quality of key life-saving interventions related to prevention, screening and management of maternal and newborn complications. As part of these surveys, health providers’ screening for and management of severe pre-eclampsia and eclampsia (PE/E), maternal sepsis, postpartum hemorrhage, prolonged and obstructed labor, and newborn asphyxia were assessed using standardized checklists based on WHO guidelines [15]. Study findings have been published that address newborn care practices across six countries [16]; prevention and management of postpartum hemorrhage (PPH) in six countries [17]; respectful mother care across five countries [18]; newborn care in Tanzania [19]; and quality of care for PPH and PE/E in Madagascar [20].

This paper presents findings on the quality of clinical practices for the identification and management of PE/E in antenatal and labor and delivery care in six sub-Saharan Africa countries: Ethiopia, Kenya, Madagascar, Mozambique, Rwanda and Tanzania (including Zanzibar). These findings represent unique information, as direct observations on the identification and management of PE/E in multiple countries are not currently described in the literature. The findings in this paper provide important information on the quality of PE/E services in multiple countries and levels of facility to identify gaps in care, and can be used by Ministries of Health and other stakeholders to improve lifesaving health services for women with PE/E.

## Methods

### Study Design

The study comprised cross-sectional QoC health facility assessments in Ethiopia, Kenya, Madagascar, Mozambique, Rwanda, and Tanzania, including Zanzibar, conducted at different times from 2010 to 2012. Zanzibar results are presented separately from the rest of Tanzania since an independent survey was conducted there.

The objectives of the QoC health facility assessments with respect to PE/E were to assess the frequency and quality of adherence to WHO standards for screening and management of PE/E, based on the 2000 WHO Management of Complications in Pregnancy and Childbirth (MCPC) reference manual [15]. These included: 1) the frequency and quality of screening for pre-eclampsia during ANC and L & D services; 2) health worker compliance with the WHO standards of management of women with severe PE/E; 3) availability of facility supplies and equipment for screening and management of PE/E; and 5) availability of national policies and guidelines for screening and management of PE/E.

### Sampling

A multi-stage sample was drawn whose primary consideration was the number of direct observations of women during labor and delivery. This started with a sampling frame of eligible facilities. A sample was drawn from a line listing of health eligible facilities, stratified by facility type (hospital/health center). The eligible facilities from which the sample was drawn varied by country based on the specific objectives of the study in that country, which were decided in consultation with national Ministries of Health and other stakeholders. In each case, the sample was two-stage, with probability proportional to utilization of maternity services. In the first stage, a list of eligible facilities within the target geographic area was compiled and organized by the number of annual deliveries. Either all hospital-level facilities that met the country study criteria were selected if the number was small (Ethiopia, Madagascar, Rwanda, Tanzania) or a random sample was drawn from this list (Kenya, Mozambique). A small sample of lower level facilities was also selected (with the exception of Ethiopia, where lower level facilities were not included). Lower level facilities in Tanzania were oversampled as part of a project evaluation design. While in most countries a nationally representative sample of women giving birth in hospitals and a small group of lower level facilities was sought (Kenya, Madagascar, Mozambique, Rwanda), in Tanzania the study was conducted as an evaluation of health facilities in 11 regions participating in a quality improvement program.

The sample size was calculated to generate point estimates of the quality of routine maternal and newborn practices during antenatal and labor and delivery care. Women admitted for emergency cesarean section were not observed. Since many of the key indicator baseline values for routine delivery care were not known (e.g., active management of the third stage of labor and newborn thermal care) values were assumed to be 50%, in order to generate the largest sample size. The number of deliveries observed in each facility was proportional to its delivery volume.

ANC consultations and labors and deliveries were observed at each health facility during a one – four day data collection period. The number of deliveries to be observed was determined using a design effect of 2 and precision (95% CI) of the point estimates as well as the percent change detectable from a baseline of 50% with 80% power and 95% precision.

Complicated cases (post-partum hemorrhage, severe pre-eclampsia or eclampsia, resuscitation of asphyxiated newborn) were observed in the study as they occurred. There was no sampling of complications in maternal or newborn care since these were rare events. The observations of complications was thus not designed to be generalizable, but provides an opportunity to examine observed rare events in the facility setting.

### Definitions of Key Measures

The tools used to collect data for the QoC studies are available online. We used the following definitions in assessing clinical practice and analyzing results:

**BP with proper technique**: BP with proper technique was observed on the ANC Observation Checklist [21] as well as the L & D Observation Checklist [21]. This was defined as:

- Q108 on ANC Observation; Q116 in L & D Observation: Takes blood pressure; a) takes client’s blood pressure in sitting or lateral position, and b) Takes blood pressure with arm at heart level

**Screening for PE/E in ANC services:** Health workers were observed conducting PE/E screening tasks in ANC consultations using the ANC Observation Checklist [21]. (Additional File 1) The questions analyzed included:

- A105: (History taking) Did the health worker or client discuss any of the following complications for prior pregnancies? (includes high BP; convulsions)
- A106: (History taking) Did the health worker ask about or the client mention any of the following for current pregnancy? (includes severe headaches and/or blurred vision; severe difficulty breathing; swollen face or hands; convulsions or loss of consciousness)
- Q108–03 on ANC Observation: Examine hands for edema
- A117: Did the health worker counsel the client on any of the following reasons to seek immediate medical care? (includes seek immediate care if she has convulsions; if she has severe headaches with blurred vision; if she has fast or difficult breathing)

**Testing Urine:** Testing urine for protein was inquired about on the ANC Observation Checklist and the L & D Observation Checklist [21]. This was defined as:

- Q108A-04 on ANC Observation Checklist: Perform or refer for urine test (includes test for proteinuria, bacteruria, glucose)
- Q118 on L & D Observation Checklist: Tests urine for presence of protein

**Screening for PE/E and management of PE/E in L & D:** The L & D Observation Checklist was used to observe health workers’ screening and management of PE/E L & D [21]. (Additional File 2) The questions analyzed included:

- Q105: (History Taking) Health worker asks whether the woman has experienced any of the following for current pregnancy (includes severe headaches and/or blurred vision; severe difficulty breathing; swollen face or hands; convulsions or loss of consciousness)
- Q111: (History Taking) Asks about complications during previous pregnancies (includes convulsions, high blood pressure)
- Q116: Takes blood pressure; when taking BP a) takes client’s blood pressure in sitting or lateral position and b) Takes blood pressure with arm at heart level
- Q127: Cause of referral (includes PE/E)
- Section 8: Observation of Management of PE/E (complete checklist of management of obstetric case of PE/E)

**Facility Readiness to Prevent and Manage PE/E:** The facility assessment of readiness for PE/E was conducted via interview with the facility in-charge as well as a visual observation of the presence of drugs and supplies [22]. The questions analyzed for the assessment of facility readiness included:

- F204a: Does this facility ever provide parenteral anticonvulsants for pregnancy-related hypertension?
- F210a: Does this facility ever perform caesarean sections? Injectable diazepam present in delivery area? Injectable magnesium sulfate present in delivery area? Are there guidelines for emergency obstetric care present in the delivery area?
- F304: Is taking blood pressure regularly practiced?
- F305: Is urine testing for protein regularly practiced?

### Study Procedures

Data collection tools included clinical observation checklists for ANC and L & D, management of severe PE/E, ANC and L & D service delivery area supply and equipment inventory checklist, and an interview guide and knowledge test (results not presented) for health workers in ANC and L & D. Observation checklists were based on WHO Managing Complications in Pregnancy and Childbirth (MCPC), Edition 1 [15] and informed by tested and validated tools used in the Service Provision Assessment (SPA) [23]. The L & D observation checklist was structured around stages of delivery. The checklist comprised sections specific to admission to the labor ward; management of the active phase of first stage of labor; birth; and immediate postpartum and newborn care. Each section could be observed independently of the others.

The facility supply and equipment audit tool used the Emergency Obstetric and Newborn Care needs assessment surveys [24] and the SPA as guiding documents.

Data collectors were health workers (nurses, midwives and doctors) who were given update trainings by consultant obstetricians on ANC, normal labor and birth and basic emergency obstetric and newborn care. They also participated in a one-week training on study methodology, research ethics, and orientation to the study tools and mobile data collection devices, including a practicum session in a health facility not included in the study sample. Inter-rater reliability was assessed during the training to ensure standardization of clinical observation skills among data collectors. During the training, discussion was held on what to do if an intervention was needed in order to save the life of the mother or newborn. Observers were instructed to contact other health workers in the facility and to not intervene unless necessary for the survival of the mother or newborn.

Teams of trained data collectors visited health facilities for one to four days. Six antenatal care visits were observed by the data collectors during this timeframe. All women admitted to labor and delivery for a defined period, from one to four days, were observed, depending on the volume of services. Observations of the active phase of first stage of labor were intermittent and observations of the second and third stages of labor were continuous. Data collectors directly observed labors and deliveries during a 16-h window each day that they were in a facility, including the day and evening shifts. Data collectors took eight-hour shifts so that there was one present for each shift. In high volume facilities, as many women as could be feasibly observed were included, with the rule that no more than three labors or deliveries could be observed by one data collector at the same time. Data collectors observed the management of women with PE/E though the number of observations was limited, as this was a relatively rare event.

In all countries but Kenya, data collectors entered data on ANC consultations and on women in labor and delivery directly into HTC Smart Phones or Samsung Galaxy Tablets with Mobile Data Studio software, which were pre-populated with the data collection forms. In Kenya, data were captured on paper as part of the Service Provision Assessment.

It is important to note that the sampling strategy in all country assessments was similar in use of number of deliveries as the central sampling strategy, and that the number, level and location of health facilities included in the samples varied considerably among countries based on priority settings of the Ministries. In no country was the study sampled to detect differences among levels of health facilities. We present findings on differences in service delivery components among levels of facilities as indicative rather than sampled findings.

### Analysis and Weighting

Data were transferred directly from mobile devices via cellular network to a password protected online site with pre-programmed data tables with frequency runs in SQL server. Additional descriptive statistics, including means and cross tabulations, were calculated using Microsoft Excel 2010 and IBM SPSS Statistics version 20 (Armonk, NY). The analysis of PE/E screening in L & D was done on the observations of admission to the labor and delivery ward. Qualitative, descriptive analysis of management of women with severe pre-eclampsia or eclampsia was conducted due to the small number of these events.

Weighting of data was conducted for labor and delivery observations only: observations were weighted using facility weights to adjust for over-representation as well as under-representation of facilities and, thus, of observations in the sample. The expected number of observations per day for each facility was calculated based on yearly estimates supplied by the Ministries of Health. Data for ANC observations were not weighted.

## Results

Across the six countries, 643 health facilities and 1057 health workers were included in the QoC studies. In the course of the studies, 2920 women receiving antenatal care and 2689 women in labor and delivery at the health facilities were observed. This included 1804 women observed undergoing their initial assessment during admission into the labor and delivery ward (Table 1).

**Table 1:** Facilities and participants in QoC health facility surveys, 2010–2012

Sample	Kenya	Ethiopia	Tanzania	Zanzibar	Rwanda	Madagascar	Mozambique	Total
All study facilities	409	19	52	9	72	36	46	643
Hospital	52%	100%	23%	56%	58%	75%	46%	53%
Health Center/dispensary	48%	0%	77%	44%	42%	25%	54%	47%
Total deliveries observed	626	192	489	217	293	347	525	2689
Initial assessments observed	452	107	306	106	187	268	378	1804
ANC consults observed	1409	126	391	57	311	323	303	2920

### Screening for PE/E in ANC

Asking women about danger signs in ANC consultations was generally poorly performed by health workers, with 27% of ANC clients asked about headache or blurred vision, the same number asked about swollen hands or face, and 39% asked about either danger signs (Table 2). Blood pressure (BP) assessment in ANC consultations was relatively higher, with 68% of ANC clients having their BP taken with proper technique (range 46% [Rwanda] to 96% [Kenya]). Approximately one third (31%) of ANC clients were asked about at least one danger sign **and** had their BP taken correctly (Table 2). Roughly half (46%) either had a urine test for protein performed or were referred for a urine test.

**Table 2:** Pre-eclampsia screening during antenatal care from observations of ANC consultations

	Kenya	Ethiopia	Tanzania	Zanzibar	Rwanda	Madagascar	Mozambique	Total
	*N* = 1409	*N* = 126	*N* = 391	*N* = 57	*N* = 311	*N* = 323	*N* = 303	*N* = 2920
Ask about headache or blurred vision	23%	32%	25%	51%	14%	32%	12%	27%
Ask about swollen hands or face	24%	16%	22%	35%	26%	39%	26%	27%
Asks about either sign	30%	38%	33%	60%	30%	47%	31%	39%
Take client’s BP with proper technique	96%	89%	65%	81%	46%	48%	48%	68%
Both PE/E screening elements (ask about at least 1 danger sign and take BP with proper technique)	29%	32%	24%	55%	22%	25%	25%	31%
Perform or refer for urine test	59%	66%	40%	86%	31%	29%	9%	46%

**Fig. 1:**
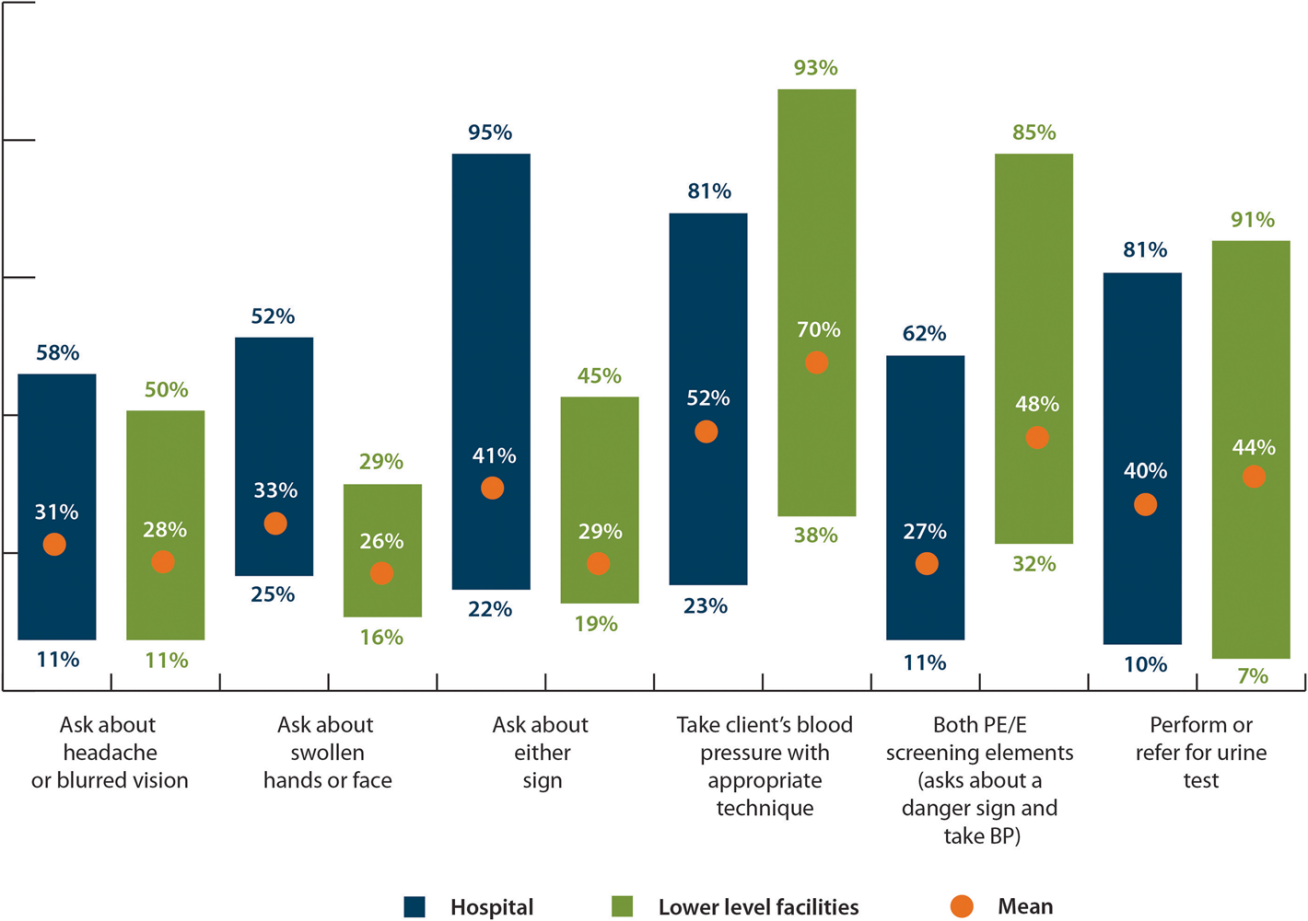
Means and range of PE/E screening among ANC consultations by level of health facility (*n* = 2920)

There were no consistent differences in PE/E screening tasks in ANC by level of facility: regardless of level, roughly one third of ANC clients were asked about dangers signs of PE/E (Fig. 1). Similarly, urine testing did not vary between levels (44% of hospital consultations and 40% of health center/dispensary consultations). The screening task which varied the most among health facility levels was correctly taking clients’ BP. Lower level health facilities on average had a higher proportion of ANC clients having their BP taken correctly (52% in hospitals compared to 70% in health centers/dispensaries).

### Screening for PE/E in L & D services

Across all study facilities, less than a quarter (24%) of women admitted to labor and delivery services were asked about signs of PE/E, ranging from 11% (Mozambique) to 34% (Kenya, Ethiopia) (Table 3). A much higher proportion of women (77%) had their BP checked upon admission, ranging from 59% (Mozambique) to 85% (Ethiopia, Zanzibar). Seven percent of women had their urine tested for the presence of protein.

**Table 3:** Pre-eclampsia screening at labor and delivery services from observations of admission to L & D

	Kenya	Ethiopia	Tanzania	Zanzibar	Rwanda	Madagascar	Mozambique	Total
	*N* = 452	*N* = 107	*N* = 306	*N* = 106	*N* = 187	*N* = 268	*N* = 378	*N* = 1804
Asks about signs of PE/E	34%	34%	14%	25%	16%	29%	11%	24%
Initial blood pressure check	71%	85%	83%	85%	73%	82%	59%	77%
Both PE/E screening elements	20%	29%	14%	26%	15%	29%	8%	20%
Tests urine for presence of protein	12%	8%	3%	12%	4%	7%	2%	7%
BP recorded at least every 4 h (when diastolic < 90 mmHg)	47%	8%	37%	29%	50%	17%	15%	29%

Some variation was observed in screening for PE/E in L & D between hospitals and lower level health facilities: 82% of women admitted to L & D services in hospitals and 67% of women admitted to L & D services in lower-level health facilities had their BP checked upon admission. Urine testing was uniformly low at 7% in both levels.

### Management of Severe PE/E

**Fig. 2:**
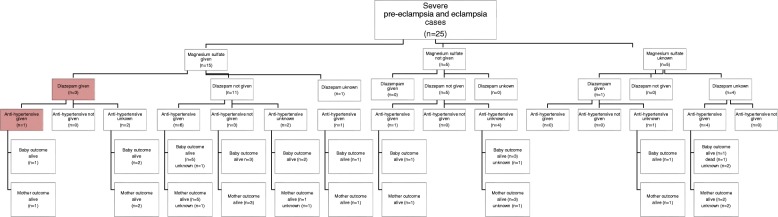
Management of Severe PE/E Cases (*n* = 25)

Across the six countries, management of 25 cases of severe PE/E was observed (Fig. 2). These cases are presented in Fig. 2 according to whether MgSO4 was given (*n* = 15), not given (*n* = 5), or this information was unknown (*n* = 5). The three cases in which both MgSO4 and diazepam were given are highlighted in red as this is not recommended by WHO guidelines and is a potentially dangerous combination. In at least 5 cases (5/13), the woman didn’t receive anti-hypertensive, although this is a standard of care for all cases of PE/E. In the 25 cases observed, no mothers treated for PE/E died but one newborn did not survive (indicated in box). In this case, it was unknown whether the mother received MgSO4 or diazepam, but she did receive an anti-hypertensive medication. Our data do not provide enough information to determine the cause of the newborn death.

### Facility Readiness to Provide PE/E Screening and Management

Magnesium sulfate was included in the essential drug list in all survey countries at the time of the study. However, based on the facility audit, availability of the drug at the study sites varied widely (Table 4). In Ethiopia, 16% of the L & D wards in study facilities had MgSO4, in Madagascar 55%, and in Mozambique MgSO4 was available in the L & D ward of all of the surveyed facilities on the day of the survey. Availability of MgSO4 in health centers similarly varied, ranging from 4% (Rwanda) to 96% (Mozambique).

**Table 4:** Health Facility Readiness: Availability of Supplies and equipment for management of and screening for PE/E

	Kenya	Ethiopia	Tanzania	Zanzibar	Rwanda	Madagascar	Mozambique
Labor and Delivery Area	*N* = 171	*N* = 19	*N* = 52	*N* = 9	*N* = 72	*N* = 36	*N* = 41
MgSO4 available							
Hospital	72%	16%	83%	60%	70%	55%	100%
Health Center/Dispensary	57%	–	35%	75%	4%	17%	96%
Total	70%	16%	47%	67%	43%	46%	98%
	Kenya	Ethiopia	Tanzania	Zanzibar	Rwanda	Madagascar	Mozambique
Antenatal Care Area	*N* = 409	*N* = 19	*N* = 52	*N* = 9	*N* = 72	*N* = 36	*N* = 41
% of health facilities with urine test strips or ability to do urine test	N/A	N/A	20%	75%	26%	29%	21%
% of study health facilities with functioning BP apparatus is available	N/A	100%	96%	100%	92%	82%	70%

## Discussion

This study assessed screening for PE/E in both ANC and labor and delivery services, management of a 25 cases of severe PE with MgSO4 and anti-hypertensives, using observations of actual care, from six SSA countries. Additionally, a facility readiness assessment audited availability of MgSO4, anti-hypertensives, guidelines for management of obstetric complications and BP equipment for provision of PE/E screening and management.

The observed ANC consultations showed a low level of provision of WHO-recommended PE/E screening practices in ANC services, with only one third (31%) of the ANC clients receiving the recommended screening. This is not dissimilar to other studies which have looked at screening services during ANC Findings from Zambia suggest that 83% of women attending ANC had their BP checked [25]. According to a WHO analysis of risk factors for PE/E, having more than 8 ANC visits is protective, presumably because of more frequent inquiries about danger signs and blood pressure measurements [2]. This underlines the importance of addressing gaps in quality of antenatal care.

Findings from the L & D observations indicated that screening for PE/E was also low, with less than a quarter (23%) of women admitted to L & D services asked about signs of PE/E. Seventy-seven percent of women admitted to L & D services had their BP checked, but this proportion was as low as 59% in one country (Madagascar). These data indicate that there are many missed opportunities to identify and manage women with pre-eclampsia as early as possible to prevent maternal and newborn complications.

Current (2015) guidance recommends testing for proteinuria as part of screening for PE/E if the diastolic BP is ≥90 mmHg. In the facilities in our study, urine testing was very uncommon among women admitted to maternity services (7%). This is even lower than findings from Zambia where 23% of health facilities conducted urine tests [25]. The low level of urine testing in L & D may represent multiple factors that our study was not able to distinguish. These include supply chain management issues leading to stock-outs of supplies; absence of clear, up-to-date national guidelines, and/or low health worker understanding of and compliance with guidelines on urine protein testing. It is strongly recommended that these issues be identified and addressed by facility administrators and other stakeholders so that women receive the evidence-based care that will help reduce PE/E-related morbidity and mortality.

This study presents the only known documentation of assessment of care of women with severe pre-eclampsia/eclampsia by direct observation using standardized tools based on WHO guidelines. The 25 cases observed cannot be used to draw generalizable conclusions. Still, within these 25 cases it was noted that only 15 women diagnosed with severe PE/E received MgSO4, and in three of those cases the women were treated with both MgSO4 and diazepam, a potentially dangerous practice which is not based on global recommendations. In addition, 13 women received anti-hypertensives while five did not (information was missing in nine cases). While these findings cannot be generalized, they are illustrative of quality of care issues associated with management of severe PE/E which directly relate to maternal and newborn survival.

Although MgSO4 has been recommended for use in management of PE/E by WHO [5], the uptake of this drug for management of PE/E has been fraught with difficulties in many countries. These include absence of national-level policy guidance; lack of registration of the drug at national level; low incentive for pharmaceutical companies to manufacture and sell the drug due to poor profit margins; poor distribution systems which cause stock-outs; and uncertainty by health workers about the safety profile and use of the drug. An assessment conducted in twelve countries concluded that barriers to use of MgSO4 are varied and tend to be regional, such as negative provider perceptions and resulting low utilization, policies from Ministries (MgSO4 use for management of eclampsia but not severe pre-eclampsia, for use at higher level health facilities only), pharmaceutical companies undervaluing the drug because of its low price, and poor supply and availability of MgSO4 in health facilities [26]. These issues were described in Pakistan, where low use is related to provider perception of the safety of the drug, despite a national policy that approves its use [27]. In Mozambique, Zimbabwe and Zambia, health worker concerns about safety and low utilization of the drug were described [28, 29]. Because MgSO4 is very inexpensive, there is a lack of financial incentive for drug manufacturers to push forward registration; this was apparent as a barrier in Zambia in 2005, where the drug was not registered and there was no MOH policy recommending use of the drug [29].

All of the barriers described in the literature were likely reflected in the findings of our study. In Rwanda and Ethiopia, at the time of the assessment, MOH policy did not allow MgSO4 use at lower level facilities. This could be related to health workers perceptions. A study conducted in Pakistan found that health care workers (in particular older generation physicians), feel that using MgSo4 outside facilities with intensive care units is unsafe [27]. Availability of MgSO4 in the labor and delivery areas of the study facilities varied from 16% (Ethiopia) to 98% (Mozambique). While some countries had strong availability, most of the countries assessed had less than optimal access to this life-saving drug for management of PE/E as an obstetric emergency.

As evidence continues to emerge that increasing access to and utilization of maternity care in a facility setting does not automatically translate into better maternal outcomes, quality of maternal health care is increasingly important [30]. Approaches to measurement of quality of care that are effective, accurate and provide relevant information become increasingly important. The studies described in this article were among a very few which utilized direct observation of services as a methodology to assess quality of care.

### Limitations

While results for six countries are presented here, the QoC studies in each country were conducted separately rather than as a multi-country study. All countries used the same tools, with minor modification to fit national policy. However, the sampling frameworks, as well as which facilities were assessed, were informed by nationally driven study priorities for quality improvement. Thus, although the sampling approach to delivery observations was comparable, the approach to sampling health facilities across countries varied substantially. The results must be viewed with these sampling differences in mind.

In none of the countries was the study sampled to compare management of care between lower level facilities and hospitals. Thus our presentation of results comparing lower level facilities and hospitals is observational and based on available data rather than sampled data. The differences in provision of care between lower level facilities and hospitals should be viewed as indicative.

The observational findings may be subject to some level of bias due to the Hawthorne effect. The study design attempted to mitigate this by having data collection teams stay for multiple days, which may have helped to reduce health worker awareness of the presence of a data collector. While we were unable to characterize this type of bias in our study results, the presented data could be viewed as the best possible care that the providers could or did provide.

Some aspects of PE/E screening are difficult to observe, particularly asking about swelling of the face and hands, as a health worker may perform this task but not ask the client, which could result in underreporting of this aspect of screening. Finally, the management of the 25 cases of severe PE observed were not sampled, rather these occurred in the health facilities during the data collection period. The objective of the study in relation to management was to assess only the initial management tasks called “stabilization” (MgSo4 & anti hypertensives). Observation use of gestational age to inform timing of delivery’ was not included in the study. The information about management of these cases is not presented as a basis to draw inferences about management of all PE/E cases in the participating countries; rather it can serve as a basis to probe further about the concerns raised during these observations.

### Conclusions and Recommendations

This study highlighted notable deficiencies in the six study country health services in relation to PE/E screening and treatment, which are echoed in findings from the literature. Findings from these studies suggest that the majority of women attending ANC and L & D services are not being screened according to WHO-recommended standards. Lowest performance related to assessment of presence of danger signs, both at ANC visits and upon admission to L & D services. Additionally, for management of PE/E, our findings on MgSO4 availability indicate that supplies and usage of MgSO4 in L & D may be inadequate. Given WHO guidance on this issue, policy makers in these countries are urged to take policy and practice-related steps to increase availability and use of MgSO4 in L & D services. Illustrative findings on treatment in women with severe PE/E highlighted problematic areas of usage of MgSO4 and non-use of indicated anti-hypertensives which should be further investigated. Future research could better understand constraints as well as facilitators to providing appropriate PE/E screening and treatment, including national policy factors and health worker attitudes and behavior. However, it is clear that there are gaps in service delivery quality in both ANC and L & D that could be addressed immediately to improve access to this life-saving intervention.

## Additional files

Additional file 1: Maternal and Newborn Quality of Care Survey ANC Observation Checklist. The standardized data collection tool which was modified for use in each country for data collection for ANC services. (PDF 163 kb)

Additional file 2: Maternal and Newborn Quality of Care Survey L & D Observation Checklist. The standardized data collection tool which was modified for use in each country for data collection for labor and delivery services. (PDF 285 kb)

## Abbreviations

AMDD: Averting Maternal Death and Disability
ANC: Antenatal Care
BP: Blood Pressure
L & D: Labor and Delivery
MCHIP: Maternal and Child Health Integrated Program
MCPC: Management of Complications in Pregnancy and Childbirth
PE/E: Preeclampsia and Eclampsia
SARA: Service Availability and Readiness Assessments
WHO: World Health Organization
