# Supplementary material for: Screening and management of pre-eclampsia and eclampsia in antenatal and labor and delivery services: findings from cross-sectional observation studies in six sub-Saharan African countries
Source: BMC Pregnancy Childbirth. 2018 Aug 23;18:346. doi: 10.1186/s12884-018-1972-1 (PMC6108136; doi:10.1186/s12884-018-1972-1)
Supplement: Supplementary file 1 — Maternal and Newborn Quality of Care Survey ANC Observation Checklist. The standardized data collection tool which was modified for use in each country for data collection for ANC services. (PDF 163 kb) [file 12884_2018_1972_MOESM1_ESM.pdf]

Maternal and Newborn Quality of Care Survey

ANC Observation Checklist

Cover Page	
A1: Facility name	A2: Facility number
A3: Observer number	A4: Today's date (day/month/year)

FIND A HEALTH WORKER INVOLVED IN ANTENATAL CARE SERVICES. IF THIS IS A NEW RESPONDENT, OBTAIN INFORMED CONSENT BELOW. IF THE PERSON IS NOT A NEW RESPONDENT, PROCEED TO A5. BEFORE OBSERVING THE CONSULTATION, MAKE SURE TO OBTAIN PERMISSION FROM BOTH THE SERVICE PROVIDER AND THE CLIENT. ALSO MAKE SURE THAT THE PROVIDER KNOWS THAT YOU ARE NOT THERE TO EVALUATE HIM OR HER, AND THAT YOU ARE NOT AN "EXPERT" TO BE CONSULTED DURING THE SESSION.

READ ORAL CONSENT SCRIPT TO HEALTH WORKER.

A5: *Ask health worker* Do I have your permission to be present at this consultation?

☐ Yes, consent is given → go to A6

☐ No, consent is not given → observation of this health worker must <u>END</u>; if available, approach another health worker for participation.

A6: Health worker line number (from staff listing)			A7: Sex of health worker	
			Male	1
			Female	2
A8: Health worker category	Category 1	1		
	Category 2	2		
		3		
		4		
		5		
		6		
		7		
		8		
		9		
		10		

READ ORAL CONSENT SCRIPT TO CLIENT.

A9: *Ask client* Do I have your permission to be present while you are receiving services today?

☐ Yes, consent is given → go to A11

☐ No, consent is not given → observation of this client must <u>END</u>; if available, approach another client for participation.

A11: Client code	*Start client code at 1 for each new facility visited.*

Maternal and Newborn Quality of Care Survey

ANC Observation Checklist

Section 1: Introduction and History Taking

RECORD WHETHER THE PROVIDER CARRIED OUT THE FOLLOWING STEPS AND/OR EXAMINATIONS:

Record the time that ANC consultation started				
**Question**	**Yes**	**No**	**DK**	**Go to**
A100: Did the health worker greet the client (and others present) in a friendly and respectful manner?	1	0	8	
A101: Did the health worker introduce her/himself and title (midwife, nurse, etc.)	1	0	8	
A102: Did the health worker call the client by her appropriate name or appropriate title?	1	0	8	
A103: Did the health worker ask about or the client mention any of the following facts?				
01) Client's age	1	0	8	
02) Medication the client is taking	1	0	8	
03) Date that client's last menstrual period began	1	0	8	
04) Prior pregnancies	1	0	8	
A104: Number of prior pregnancies *(Observer: listen and record woman's number of prior pregnancies; enter 0 if no previous pregnancies, enter 98 if number not mentioned)*				No prior pregnancies→A106
A105: Did the health worker or client discuss any of the following complications for prior pregnancies?				
01) Heavy bleeding during or after delivery	1	0	8	
02) Anemia	1	0	8	
03) High blood pressure	1	0	8	
04) Convulsions	1	0	8	
05) Multiple pregnancies (twins or above)	1	0	8	
06) Prolonged labour	1	0	8	
07) C-section	1	0	8	
08) Assisted delivery (forceps, ventouse)	1	0	8	
09) Prior neonatal death (death of baby less than 1 month old)	1	0	8	
10) Prior stillbirth (baby born dead that does not breathe or cry)	1	0	8	
11) Prior abortion/miscarriage (loss of pregnancy)	1	0	8	
A106: Did the health worker ask about or the client mention any of the following for current pregnancy?				
01) Vaginal bleeding	1	0	8	
02) Fever	1	0	8	
03) Headaches or blurred vision	1	0	8	
04) Swollen face or hands	1	0	8	
05) Convulsions or loss of consciousness	1	0	8	
06) Severe difficulty breathing	1	0	8	
07) Persistent cough for 2 weeks or longer	1	0	8	
08) Severe abdominal pain	1	0	8	
09) Foul smelling discharge	1	0	8	
10) Frequent or painful urination	1	0	8	
11) Whether the client has felt a decrease or stop in fetal movement	1	0	8	
12) If there are any other problems the client is concerned about	1	0	8	

END OF SECTION 1

Maternal and Newborn Quality of Care Survey

ANC Observation Checklist

Section 2: Tests and Treatments				
**Question**	**Yes**	**No**	**DK**	**Go to**
A107: Did the health worker wash his/her hands with soap or use alcohol hand rub prior to examination?	1	0	8	
A108: Did the health worker perform any of the following procedures?				
01) Weigh the client	1	0	8	
02) Take the client's blood pressure	1	0	8	No/DK→A108_03
02a) Take client's blood pressure in sitting or lateral position	1	0	8	
02b) Take blood pressure with arm at heart level	1	0	8	
03) Examine hands for edema	1	0	8	
04) Perform or refer for urine test	1	0	8	No/DK→A108_05
04a) Test for proteinuria	1	0	8	
04b) Test for bacteriuria	1	0	8	
04c) Test for glucose	1	0	8	
05) Check for signs of anemia	1	0	8	
06) Perform or refer for anemia test	1	0	8	
07) Palpate the client's abdomen for uterine height	1	0	8	
08) Listen to the client's abdomen for fetal heartbeat	1	0	8	
09) Perform or refer for a syphilis test	1	0	8	
A109: Did the health worker ask about or the client mention her HIV status?	1	0	8	
A110: Did the health worker perform, inquire about, or refer for an HIV test?	1	0	8	
A111: Is client HIV positive? *(Observer: listen and record answer, circle Don't Know if HIV status is unknown or status is not discussed)*	1	0	8	
A112: Did the health worker provide any counseling on HIV/PMTCT?	1	0	8	No/DK→A114
A113: Did the health worker provide counseling on the following HIV/PMTCT topics?				
01) Explain the purpose of ARV prophylaxis	1	0	8	
02) Explains when to collect NVP	1	0	8	
03) Explains when to take NVP at the onset of labour	1	0	8	
04) Explains how to take AZT at 14 weeks	1	0	8	
05) Explains the advantages and side effects of ART	1	0	8	
06) Explains feeding options for exposed babies	1	0	8	
07) Explains about importance of bringing exposed infant back for testing	1	0	8	
A114: Did the health worker refer woman to CTC?	1	0	8	
A115: Did the health worker give the client any of the following treatments?				
01) Prescribed iron or folic acid (IFA) or both	1	0	8	
02) Gave supply of iron or folic acid (IFA) or both	1	0	8	If A115_01 AND A115_02 are No/DK→A115_06
03) Explained the purpose of iron or folic acid	1	0	8	
04) Explained how to take iron or folic acid pills/syrup	1	0	8	
05) Explained side effects of iron or folic acid	1	0	8	
06) Prescribed or gave a tetanus toxoid (TT) injection	1	0	8	No/DK→A115_08
07) Explained the purpose of the TT injection	1	0	8	
08) Prescribed or gave anti-malarial prophylaxis (SP for IPT)	1	0	8	No/DK→A115_12
09) Explained the purpose of the preventative treatment with anti-malarial medication	1	0	8	
10) Explained how to take the anti-malarial medication	1	0	8	
11) Explained side effects of anti-malarial medication	1	0	8	
12) Gave voucher for ITN or gave free ITN, or ITN already purchased by client	1	0	8	
13) Importance of using ITN explained explicitly	1	0	8	
14) Prescribed or gave deworming medication	1	0	8	No/DK→A116
15) Explained the purpose of deworming	1	0	8	
16) Explained how to take deworming medication	1	0	8	
17) Explained side effects of deworming medication	1	0	8	
**END OF SECTION 2**				

Section 3: Counseling and Outcome

Question	Yes	No	DK	Go to
A116: Did the health worker inform the client about the progress of the pregnancy?	1	0	8	
A117: Did the health worker counsel the client in any of the following reasons to seek immediate medical care?				
01) Seek immediate care if she has vaginal bleeding	1	0	8	
02) If she has convulsions	1	0	8	
03) If she has severe headaches with blurred vision	1	0	8	
04) If she has fever and is too weak to get out of bed	1	0	8	
05) If she has severe abdominal pain	1	0	8	
06) If she has fast or difficult breathing	1	0	8	
A118: Did the health worker counsel the client in any of the following ways about birth preparation?				
01) Asked the client where she will deliver	1	0	8	
02) Advised the client to prepare for delivery (e.g. set aside money, arrange for emergency transportation)	1	0	8	
03) Advised the client to use a skilled health worker during delivery	1	0	8	
04) Discussed with client what items to have on hand at home for emergencies (e.g. sterile blade)	1	0	8	
A119: Did the health worker discuss nutrition and healthy eating during pregnancy?	1	0	8	
A120: Did the health worker discuss breastfeeding?	1	0	8	
A121: Did the health worker discuss family planning for use after delivery?	1	0	8	
A122: Did the health worker counsel on when to return for next visit?	1	0	8	
A123: Did the health worker ask whether the client had any questions?	1	0	8	
A124: Did the health worker use any visual aids for health education or counseling during the consultation?	1	0	8	
A125: Did the health worker speak using easy-to-understand language for the client?	1	0	8	
A126: Did the health worker look at the client's health card/booklet, either before beginning the consultation or while collecting information or examining the client (*Observer: choose DK if no card/booklet*)	1	0	8	
A127: Did the health worker write on the client's health card? (*Observer: choose DK if no card/booklet*)	1	0	8	
Record the time ANC consultation ended				

REMEMBER TO THANK CLIENT FOR THEIR PARTICIPATION IN THE STUDY

AT THE END OF THE CONSULTATION, ASK THE HEALTH WORKER THE FOLLOWING QUESTIONS:

A128: Ask the health worker how many weeks pregnant the client is (*Observer: enter 98 for Don't Know)*		
**Question**	**Code**	
A129: Ask the health worker whether this is the client's 1st, 2nd, 3rd, 4th, or 5th visit for antenatal care at this facility for this pregnancy		
• First visit	1	
• Second visit	2	
• Third visit	3	
• Fourth visit	4	
• Fifth or more visit	5	
A130: Ask the health worker whether this is a referral visit or a routine ANC visit		
• Referral visit	1	
• Routine visit	2	
A131: Ask the health worker whether this is the client's first pregnancy *(Observer: circle that the answer to A104_4 agrees with the answer to this question)*		
• Yes, first pregnancy	1	
• No	2	
A132: Record the outcome of the consultation (what happened at the time the observation concluded)		
Client goes home	1	→A134
Client referred (same facility)	2	
Client admitted (same facility)	3	
Client referred to other facility	4	
Don't Know	8	→A134

A133: If client was admitted or referred, ask health worker what is client's diagnosis
A134: *PLEASE COMMENT ON THE QUALITY OF CARE PROVIDED:*
**REMEMBER TO THANK PROVIDER FOR THEIR PARTICIPATION IN THE STUDY**
**END OF ANC OBSERVATION**
