# Supplementary material for: Screening and management of pre-eclampsia and eclampsia in antenatal and labor and delivery services: findings from cross-sectional observation studies in six sub-Saharan African countries
Source: BMC Pregnancy Childbirth. 2018 Aug 23;18:346. doi: 10.1186/s12884-018-1972-1 (PMC6108136; doi:10.1186/s12884-018-1972-1)
Supplement: Supplementary file 2 — Maternal and Newborn Quality of Care Survey L & D Observation Checklist. The standardized data collection tool which was modified for use in each country for data collection for labor and delivery services. (PDF 285 kb) [file 12884_2018_1972_MOESM2_ESM.pdf]

Maternal and Newborn Quality of Care Survey

L&D Observation Checklist

Q12 [ ]

Cover Page	
Facility name	Facility number
Observer number	Today's date (day/month/year)

FIND A HEALTH WORKER INVOLVED IN DELIVERY CARE SERVICES. IF THIS IS A NEW RESPONDENT, OBTAIN INFORMED CONSENT BELOW. IF THE PERSON IS NOT A NEW RESPONDENT, PROCEED TO Q5. BEFORE OBSERVING THE CONSULTATION, MAKE SURE TO OBTAIN PERMISSION FROM BOTH THE SERVICE PROVIDER AND THE CLIENT. ALSO MAKE SURE THAT THE PROVIDER KNOWS THAT YOU ARE NOT THERE TO EVALUATE HIM OR HER, AND THAT YOU ARE NOT AN "EXPERT" TO BE CONSULTED DURING THE SESSION.

READ ORAL CONSENT SCRIPT TO HEALTH WORKER.

Q5: *Ask health worker* Do I have your permission to be present at this consultation?

☐ Yes, consent is given → go to Q6

☐ No, consent is not given → observation of this health worker must END; if available, approach another health worker for participation.

Q6: Health worker line number (from staff listing)			Q7: Sex of health worker		
				Male	1
				Female	2
Q8: Health worker category	Category 1	1			
		2			
		3			
		4			
		5			
		6			
		7			
		8			
		9			
		10			

READ ORAL CONSENT SCRIPT TO CLIENT. IF CLIENT IS INCAPACITATED, NEXT OF KIN OR FAMILY FRIEND ACCOMPANYING CLIENT MAY GIVE CONSENT. CONSENT FOR CLIENT CANNOT BE GIVEN BY HEALTH WORKER OR FACILITY IN-CHARGE. CLIENT OR PROXY CONSENT MUST BE OBTAINED PRIOR TO START OF OBSERVATION.

Q9: *Ask client* Do I have your permission to be present while you are receiving services today?

☐ Yes, consent is given → go to Q10

☐ No, consent is not given → observation of this client must END; if available, approach another client for participation.

Q10: Who gave consent	Client	1	
	Next of kin/family friend	2	
Q11: Client code			*Start client code at 1 for the first client observed at a given facility.*
Q12: Client initials or other identifier			*Write client initials or identifier in box at top right of cover first page (marked Q12) to help identify this client's case when observing multiple cases*
Q13: Record time the observation started			

MOST CLIENTS WILL BE IN LABOR WHEN ADMITTED AND OBSERVATION STARTS, HOWEVER SOME CLIENTS MAY HAVE ALREADY DELIVERED AND COME TO THE FACILITY WHEN THEY ARE EXPERIENCED A COMPLICATION (PPH OR PE/E). ALSO, SOME CLIENTS MAY EXPERIENCE A COMPLICATION (PE/E) BEFORE GOING INTO LABOR AND BE TREATED IN LABOR WARD.

Q14: Client is admitted for:	Labor and delivery	1	→ ***GO TO SECTION 1 TO BEGIN THE OBSERVATION***
	Complication	2	
Q15: Where is client coming from:			
	Other health facility	1	
	ANC ward at this facility	2	
	Home/someplace else	3	
Q16: Type of complication			
	Postpartum hemorrhage	1	→ ***GO TO PPH CHECKLIST TO BEGIN THE OBSERVATION***
	Pre-eclampsia/eclampsia	2	→ ***GO TO PE/E CHECKLIST TO BEGIN THE OBSERVATION***

Maternal and Newborn Quality of Care Survey

L&D Observation Checklist

Section 1: Initial Client Assessment

Question	Yes	No	DK	Go to
Q100: Was this section observed?	1	0		No → Q200
PLEASE ANSWER Q100 BEFORE PROCEEDING: WAS THIS SECTION OBSERVED? IF SECTION NOT OBSERVED, SKIP TO NEXT SECTION.				
RECORD WHETHER THE PROVIDER CARRIED OUT THE FOLLOWING STEPS AND/OR EXAMINATIONS: (SOME OF THE FOLLOWING STEPS MAY BE PERFORMED SIMULTANEOUSLY OR BY MORE THAN ONE PROVIDER)				
**INTRODUCTION AND HISTORY TAKING**				
Q101: Respectfully greets the pregnant woman	1	0	8	
Q102: Encourages the women to have a support person present during labor and birth	1	0	8	
Q103: Asks woman (and support person) if she has any questions	1	0	8	
Q104: Checks client card OR asks client her age, length of pregnancy, and parity	1	0	8	
Q105: Asks whether she has experienced any of the following for current pregnancy:				
01) Vaginal bleeding	1	0	8	
02) Fever	1	0	8	
03) Severe headaches and/or blurred vision	1	0	8	
04) Swollen face or hands	1	0	8	
05) Convulsions or loss of consciousness	1	0	8	
06) Severe difficulty breathing	1	0	8	
07) Persistent cough for 2 weeks or longer	1	0	8	
08) Severe abdominal pain	1	0	8	
09) Whether the client has felt a decrease or stop in fetal movement	1	0	8	
10) If there are any other problems the client is concerned about	1	0	8	
Q106: Checks woman's HIV status (checks card or asks woman)	1	0	8	
Q107: Offers woman HIV test if status unknown	1	0	8	
Q108: Is woman HIV positive? *(observer: listen and record answer; circle Don't Know if status is unknown or is not discussed.*	1	0	8	No/DK → Q110
Q109: Asks about or counsels on the following topics for HIV positive mothers:				
01) Asks if client is currently taking ARVS	1	0	8	No/DK → Q109_02
02) Explains why the mother should take ARVs	1	0	8	
03) Explains when and how the mother should take ARVs	1	0	8	
04) Administers ARVs to mother	1	0	8	
05) Explains why the newborn should take ARVs	1	0	8	
06) Explains when and how newborn should take ARVs	1	0	8	
Q110: Client has any previous pregnancies? *(observer: listen and record answer)*	1	0	8	No/DK → Q112
Q111: Asks about complications during previous pregnancies:				
01) Heavy bleeding during or after delivery	1	0	8	
02) Anemia	1	0	8	
03) High blood pressure	1	0	8	
04) Convulsions	1	0	8	
05) Multiple pregnancies (twins or above)	1	0	8	
06) Prolonged labour	1	0	8	
07) C-section	1	0	8	
08) Assisted delivery (forceps, vacuum extraction)	1	0	8	
09) Prior neonatal death (death of baby less than 1 month old)	1	0	8	
10) Prior stillbirth (baby born dead that does not breathe or cry)	1	0	8	
11) Prior abortion/miscarriage (loss of pregnancy)	1	0	8	
**EXAMINATION**				
Q112: Washes his/her hands with soap and water or uses alcohol hand rub before any initial examination	1	0	8	
Q113: Explains procedures to woman (support person if present or if situation allows) before proceeding	1	0	8	
Q114: Takes temperature	1	0	8	
Q115: Takes pulse	1	0	8	
Q116: Takes blood pressure	1	0	8	No/DK → Q117
01) Take client's blood pressure in sitting or lateral position	1	0	8	
02) Take blood pressure with arm at heart level	1	0	8	
Q117: Asks/notes amount and colour of urine output	1	0	8	

Q118: Tests urine for presence of protein	1	0	8	
Q119: Performs general examination (e.g. for anemia, edema)	1	0	8	
Q120: Performs the following steps for abdominal examination:				
01) Checks fundal height with measuring tape	1	0	8	
02) Checks fetal presentation by palpation of abdomen	1	0	8	
03) Checks fetal heart rate with fetoscope/doppler/ultrasound	1	0	8	
Q121: Wash her/his hands with soap and water or uses alcohol hand rub before vaginal examination<br>Q122: Wears sterile gloves for vaginal examination<br>Q123: Informs the woman what will happen before conducting vaginal examination	1	0	8	
Q124: Performs vaginal examination	1	0	8	
Q125: Informs pregnant woman of findings	1	0	8	
Q126: Was this woman referred for a c-section	1	0		No → Q200
Q127: Cause of referral	**Code**			
Obstructed Labor	A			
Pre-eclampsia/eclampsia	B			
Placenta previa	C			
Previous c-section scar	D			
Fetal distress	E			
Cord prolapse	F			
Other	X			

END OF SECTION 1

Section 2: Intermittent Observation of First Stage of Labor

Question	Yes	No	DK	Go to
Q200: Was this section observed?	1	0		No → Q300
PLEASE ANSWER Q200 BEFORE PROCEEDING: WAS THIS SECTION OBSERVED? IF SECTION NOT OBSERVED, SKIP TO NEXT SECTION.				
RECORD WHETHER THE PROVIDER CARRIED OUT THE FOLLOWING STEPS AND/OR EXAMINATIONS: (SOME OF THE FOLLOWING STEPS MAY BE PERFORMED SIMULTANEOUSLY OR BY MORE THAN ONE PROVIDER)				
**PROGRESS OF LABOR**				
Q201: At least once, explains what will happen in labor to woman (support person if present)	1	0	8	
Q202: At least once, encourages woman to consume fluids/food during labor	1	0	8	
Q203: At least once, encourages/assists woman to ambulate and assume different positions during labor	1	0	8	
Q204: Observer: Is a support person present at some point during labor?	1	0	8	
Q205: Partograph used to monitor labor	1	0		No→ Q211
Q206: Action line on partograph reached	1	0	8	No/DK → Q211
Q207: Record time action line was reached				
Q208: If action line reached on partograph, was any <u>definitive</u> action taken?	1	0	8	No/DK → Q211
Q209: Record time action was taken				
Q210: What definitive action was taken:	**Code**			
Consult with specialist	1			
Refer to other facility for specialist	2			
Prepare for assisted delivery	3			
Prepare for c-section	4			
Other (specify____________)	6			
**EXAMINATION & PROCEDURES**				
**Question**	**Yes**	**No**	**DK**	**Go to**
Q211: Puts on clean protective clothing in preparation for birth (goggles, gown or apron)	1	0	8	
Q212: Washes his/her hands with soap and water or uses alcohol hand rub prior to any examination of woman	1	0	8	
Q213: Wears sterile surgical gloves	1	0	8	
Q214: Drapes woman (one drape under buttocks, one over abdomen)	1	0	8	
Q215: Explains procedures to woman (support person) before proceeding	1	0	8	
Q216: Number of vaginal examinations *(observer: to the best of your ability, update the answer to this question during intermittent observation of first stage of labor)*				
Q217: Augments labor with oxytocin	1	0	8	No/DK → Q218
Q218: Oxytocin administered intravenously (IV)	1	0	8	
Q219: Performs artificial rupture of membrane	1	0	8	
Q220: Administers antibiotics	1	0	8	No/DK → Q223
Q221: Why were antibiotics administered?	**Code**			
Treatment for chorioamnionitis	1			
Management of pre-labor rupture of membranes	2			
Preparation for C-section	3			
Routine/prophylactic	4			
Don't know	8			
Q222: Which antibiotic was administered? (CIRCLE ALL THAT APPLY)				
Penicillin	A			
Ampicillin	B			
Gentamicin	C			
Metronidazole	D			
Cephalosporin	E			
Other	X			
Don't know	Z			
**PREPARATION FOR DELIVERY**				
CHECK TO SEE IF THE FOLLOWING EQUIPMENT AND SUPPLIES ARE LAID OUT IN PREPARATION FOR DELIVERY. IF SOME SUPPLIES ARE IN A BIRTH KIT, LOOK/ASK TO DETERMINE WHICH ITEMS ARE INCLUDED.				
**Question**	**Yes**	**No**	**DK**	**Go to**
Q223: Prepares uterotonic drug to use for AMTSL	1	0	8	No/DK → Q225

Q224: Which drug	**Code**			
Oxytocin	1	0	8	
Ergometrine	1	0	8	
Syntometrine	1	0	8	
Misoprostol	1	0	8	
**Question**	**Yes**	**No**	**DK**	**Go to**
Q225: Timer (clock or watch with seconds hand)	1	0	8	
Q226: Self-inflating ventilation bag (250 or 500 mL)	1	0	8	
Q227: Newborn face mask size 0	1	0	8	
Q228: Newborn face mask size 1	1	0	8	
Q229: Suction bulb	1	0	8	
Q230: Catheter	1	0	8	
Q231: Suction machine	1	0	8	
Q232: At least two cloths/blankets (one to dry; one to cover)	1	0	8	
Q233: Cap/hat for the newborn	1	0	8	
Q234: Disposable cord ties or clamps	1	0	8	
Q235: Sterile scissors or blade	1	0	8	
Q236: Was this woman referred for a c-section	1	0		No → Q238
Q237: Cause of referral	**Code**			
Obstructed Labor	A			
Pre-eclampsia/eclampsia	B			
Placenta previa	C			
Previous c-section scar	D			
Fetal distress	E			
Cord prolapse	F			
Q238: Has the woman completed the first stage of labor?	1	0		Yes → Q300

IF FIRST STAGE OF LABOR IS NOT COMPLETE, CHECK ANSWERS IN THIS SECTION AGAIN 15-30 MINUTES LATER

END OF SECTION 2

Section 3: Continuous Observation of Second & Third Stage of Labor

Question	Yes	No	DK	Go to
Q300: Was this section observed?	1	0		No → Q400
PLEASE ANSWER Q300 BEFORE PROCEEDING: WAS THIS SECTION OBSERVED? IF SECTION NOT OBSERVED, SKIP TO NEXT SECTION.				
RECORD WHETHER THE PROVIDER CARRIED OUT THE FOLLOWING STEPS AND/OR EXAMINATIONS: (SOME OF THE FOLLOWING STEPS MAY BE PERFORMED SIMULTANEOUSLY OR BY MORE THAN ONE PROVIDER).				
**PREPARATION FOR DELIVERY**				
Q301: Puts on clean protective clothing in preparation for birth (goggles, gown or apron) *(yes if no contamination)*	1	0	8	
Q302: Washes his/her hands with soap and water or uses alcohol hand rub before any examination of woman *(observer: circle yes if no contamination)*	1	0	8	
Q303: Wears sterile surgical gloves *(yes if no contamination)*	1	0	8	
Q304a: Performs episiotomy if indicated	1	0	8	
Q304b: States correct indication for episiotomy	1	0	8	
Q305: Presentation of baby is cephalic (head first)	1	0	8	
**DELIVERY & UTEROTONIC**				
Q306: As baby's head is delivered, supports perineum	1	0	8	
Q307: Record time of the delivery of the baby				
Q308: Checks for another baby prior to giving the uterotonic	1	0	8	
Q309: Second baby present? *(observer: circle 1 if multiple babies)*	1	0		
Q310: Administers uterotonic?	1	0		No → Q317
Q311: Record time uterotonic given				
Q312:Timing of administration of uterotonic	**Code**			
At delivery of anterior shoulder	1			
Within 1 min of delivery of baby	2			
Within 3 min of delivery of baby	3			
More than 3 min after delivery of baby	4			
Q313: Which uterotonic given				
Oxytocin	1			
Ergometrine	2			
Syntometrine	3			
Misoprostol	4			
Q314: Record dose of uterotonic given *(observer: if necessary, ask afterwards)*				
Q315: Units of medication *(observer: if necessary, ask afterwards)*				
IU	1			
mg	2			
mL	3			
mcg	4			
Q316: Route uterotonic given:				
IM	1			
IV	2			
Oral	3			
Other	4			
Q317: Record time the cord was clamped				
**Question**	**Yes**	**No**	**DK**	
Q318: Applies traction to the cord while applying suprapubic counter traction	1	0	8	
Q319: Performs uterine massage immediately following the delivery of the placenta	1	0	8	
Q320: Was placenta delivered before administration of uterotonic? *(observer: circle Don't Know if no uterotonic was given)*	1	0	8	
Q321: Assesses completeness of the placenta and membranes	1	0	8	
Q322: Assesses for perineal and vaginal lacerations	1	0	8	
Q323: Observer: Did more than one health worker assist with the birth?	1	0		
Q324: Observer: Did mother gave birth in lithotomy position (on back)	1	0		
Q325: Observer: Is a support person (companion) for mother present at birth?	1	0		

END OF SECTION 3

Section 4: Immediate Newborn and Postpartum Care

Question	Yes	No		Go to
Q400: Was this section observed?	1	0		No → Q601
PLEASE ANSWER Q400 BEFORE PROCEEDING: WAS THIS SECTION OBSERVED? IF SECTION NOT OBSERVED, SKIP TO NEXT SECTION.				
RECORD WHETHER THE PROVIDER CARRIED OUT THE FOLLOWING STEPS AND/OR EXAMINATIONS: (SOME OF THE FOLLOWING STEPS MAY BE PERFORMED SIMULTANEOUSLY OR BY MORE THAN ONE PROVIDER)				
**IMMEDIATE CARE**				
Q401: Immediately dries baby with towel	1	0	8	
Q402: Discards the wet towel	1	0	8	
Q403: Is the baby breathing or crying?	1	0		No → Q500
IF BABY IS NOT BREATHING OR CRYING, GO TO RESUSCITATION CHECKLIST (SECTION 5)				
Q404: Places baby on mother's abdomen "skin to skin"	1	0	8	
Q405: Covers baby with dry towel	1	0	8	
Q406: If not placed skin to skin, wraps baby in dry towel	1	0	8	
Q407: Ties or clamps cord when pulsations stop, or by 2-3 minutes after birth (not immediately after birth)	1	0	8	
Q408: Cuts cord with sterile blade or sterile scissors	1	0	8	
Q409: Observer: Is a support person (companion) for mother present?	1	0		
**HEALTH CHECK**				
Q410: Checks baby's temperature 15 minutes after birth	1	0	8	
Q411: Checks baby's skin color 15 minutes after birth	1	0	8	
Q412: Takes mother's vital signs 15 minutes after birth	1	0	8	
Q413: Palpates uterus 15 minutes after delivery of placenta	1	0	8	
**FIRST HOUR AFTER BIRTH**				
Q414: Mother and newborn kept in same room after delivery (rooming-in)	1	0	8	
Q415: Baby bathed within the first hour after birth	1	0	8	
Q416: Baby kept skin to skin with mother for the first hour after birth	1	0	8	
Q417: observe breastfeeding initiated within the first hour after birth	1	0	8	
Q418: Provides tetracycline eye ointment 1% prophylaxis	1	0	8	
Q419: Administers Vitamin K to newborn	1	0	8	
Q420: Is the mother HIV positive? *(observer: listen and record answer; circle Don't Know if status is unknown or is not discussed)*	1	0	8	No/DK → Q422
Q421: Administers ARVs to newborn	1	0	8	
Q422: Administers antibiotics to mother postpartum if indicated	1	0	8	No/DK → Q425
Q423: Why were antibiotics administered?	**Code**			
Treatment for chorioamnionitis	1			
Routine/prophylactic	2			
Third stage/postpartum procedure	3			
Don't know	8			
Q424: Which antibiotic was administered? (CIRCLE ALL THAT APPLY)				
Penicillin	A			
Ampicillin	B			
Gentamicin	C			
Metronidazole	D			
Cephalosporin	E			
Other	X			
Don't know	Z			
**CLEAN-UP AFTER BIRTH**				
RECORD WHETHER THE PROVIDER CARRIED OUT THE FOLLOWING STEPS AND/OR EXAMINATIONS: (SOME OF THE FOLLOWING STEPS MAY BE PERFORMED SIMULTANEOUSLY OR BY MORE THAN ONE PROVIDER)				
**Question**	**Yes**	**No**		**Go to**
Q425: Disposes of all sharps in a puncture-proof container immediately after use	1	0	8	
Q426: Decontaminates all reusable instruments in 0.5% chlorine solution	1	0	8	
Q427: Sterilizes or uses high-level disinfection for all reusable instruments	1	0	8	
Q428: Disposes of all contaminated waste in leak-proof containers	1	0	8	
Q429: Removes apron and wipe with chlorine solution	1	0	8	
Q430: Washes his/her hands with soap and water or uses alcohol hand rub	1	0	8	

CLEAN-UP AFTER NEWBORN RESUSCITATION				
Q431: Was there a newborn resuscitation? *(observer: check answer to Q500)*	1	0		No → Q439
Q432: Disposes of disposable suction catheters and mucus extractors in a leak-proof container or plastic bag	1	0	8	
Q433: Takes the bag and mask apart and inspects for cracks and tears	1	0	8	
Q434: Decontaminates the bag and mask in 0.5% chlorine solution	1	0	8	
Q435: Sterilizes or uses high-level disinfection for bag, valve and mask	1	0	8	
Q436: Decontaminates reusable suction devices in 0.5% chlorine solution	1	0	8	
Q437: Sterilizes or uses high-level disinfection for reusable suction devices	1	0	8	
Q438: Washes his/her hands with soap and water or uses alcohol hand rub	1	0	8	
Q439: Record time L&D observation ended				

REMEMBER TO THANK CLIENT AND PROVIDER FOR THEIR PARTICIPATION IN THE STUDY

END OF SECTION 4 - GO TO SECTION 600

Section 5: Checklist for Newborn Resuscitation				
**Question**	**Yes**	**No**	**DK**	**Go to**
Q500: Was this section observed?	1	0		No → Q600
PLEASE ANSWER Q500 BEFORE PROCEEDING: WAS THIS SECTION OBSERVED? IF SECTION NOT OBSERVED, SKIP TO NEXT SECTION.				
RECORD WHETHER THE PROVIDER CARRIED OUT THE FOLLOWING STEPS AND/OR EXAMINATIONS: (SOME OF THE FOLLOWING STEPS MAY BE PERFORMED SIMULTANEOUSLY OR BY MORE THAN ONE PROVIDER)				
Q501: Record time resuscitation started				
Q502: Clears the airway by suctioning the mouth first and then the nose	1	0	8	
Q503: Stimulates baby with back rubbing	1	0	8	
Q504: ***OBSERVER:*** does newborn starts to breathe or cry spontaneously?	1	0		Yes→Q531
Q505: Calls for help	1	0	8	
Q506: Ties or clamps cord immediately	1	0	8	
Q507: Cuts cord with sterile blade or sterile scissors	1	0	8	
Q508: Places the newborn on his/her back on a clean, warm surface or towel	1	0	8	
Q509: Places the head in a slightly extended position to open the airway	1	0	8	
Q510: Tells the woman (and her support person) what is going to be done	1	0	8	
Q511: Listens to the woman and provides support and reassurance	1	0	8	
Q512: Checks mouth, back of throat and nose for secretions, and clears if necessary	1	0	8	
Q513: Places the correct-sized mask on the newborn's face so that it covers the chin, mouth and nose (but not eyes)	1	0	8	
Q514: Checks the seal by ventilating two times and observing the rise of the chest	1	0	8	
Q515: ***OBSERVER:*** is newborn's chest rising in response to ventilation?	1	0		Yes→Q524
Q516: Checks the position of the newborn's head to make sure that the neck is in a slightly extended position (not blocking the airway)	1	0	8	
Q517: Checks mouth, back of throat and nose for secretions, and clears if necessary	1	0	8	
Q518: Checks the seal by ventilating two times and observing the rise of the chest	1	0	8	
Q519: ***OBSERVER:*** is newborn's chest rising in response to ventilation?	1	0		Yes→Q524
Q520: Checks the position of the newborn's head again to make sure that the neck is in slightly extended position	1	0	8	
Q521: Repeats suction of mouth and nose to clear secretions, if necessary	1	0	8	
Q522: Checks the seal by ventilating two times and observing the rise of the chest	1	0	8	
Q523: ***OBSERVER:*** is newborn's chest rising in response to ventilation?	1	0		
IF NEWBORN'S CHEST IS NOT RISING AFTER TWO ATTEMPTS TO READJUST, OBSERVER SHOULD CALL FOR SUPERVISOR TO INTERVENE. IF A HEALTH WORKER COMPETENT IN RESUSCITATION IS NOT AVAILABLE, OBSERVER MAY CHOOSE TO INTERVENE.				
Q524: Ventilates at a rate of 30 to 50 breaths/minute	1	0	8	
Q525: Conducts assessment of newborn breathing after 1 minute of ventilation	1	0		No→Q527
Q526: Condition of newborn at assessment	**Code**			
Respiration rate 30-50 breaths/minute and no chest indrawing	1			→Q531
Respiration rate <30 breaths/minute with severe indrawing	2			
No spontaneous breathing	3			
	**Yes**	**No**	**DK**	**Go to**
Q527: Continues Ventilation	1	0		No→Q531
Q528: Conducts assessment of newborn breathing after prolonged ventilation (10 minutes)	1	0		No→Q530
Q529: Condition of newborn at assessment	**Code**			
Respiration rate 30-50 breaths/minute and no chest indrawing	1			→Q531
Respiration rate <30 breaths/minute with severe indrawing	2			
No spontaneous breathing	3			
	**Yes**	**No**	**DK**	**Go to**
Q530: Continues Ventilation	1	0		
Q531: Record time that resuscitation actions ended (or time of death if baby died)				
Q532: Was the resuscitation successful? *(observer: circle No if newborn died)*	1	0		
Q533: Arranges transfer to special care either in facility or to outside facility	1	0	8	
Q534: Explains to the mother (and her support person if available) what happened	1	0	8	
Q535: Listens to mother and responds attentively to her questions and concerns	1	0	8	

Q536: Observer: Did you call for help or intervene during the resuscitation to save the life of newborn?	1	0
Q537: *PLEASE COMMENT ON THE QUALITY OF CARE PROVIDED:* *Was mother treated respectfully? Informed of procedures to her baby? Was the situation chaotic or calm? Were there any major delays in needed treatment? If so, for what drugs/procedures and why? Were multiple health workers involved? Who? If newborn did not survive, describe the circumstances. Was the mother counseled about the death of newborn?*		
**END OF SECTION 5 – RETURN TO SECTION 4, Q409**		

Section 6: Outcome & Review of Documentation

Question	Code			
COMPLETE THIS SECTION FOR ALL CLIENTS				
**CONDITION OF MOTHER & NEWBORN AT END OF OBSERVATION**				
RECORD THE STATUS OF MOTHER AND NEWBORN AT THE END OF FIRST HOUR AFTER BIRTH.				
Q601: Record outcome for the mother				
Goes to recuperation ward	1			
Referred to specialist, same facility	2			
Goes to surgery, same facility	3			
Referred, other facility	4			
Death of mother	5			
Don't know	8			
Q602: Record outcome for the newborn or fetus				
Goes to normal nursery	1			
Referred to specialist, same facility	2			
Referred, other facility	3			
Goes to ward with mother	4			
Newborn death	5			
Fresh stillbirth	6			
Macerated stillbirth	7			
Don't know	8			
**POTENTIALLY HARMFUL PRACTICES**				
Q603: Did you see any of the following harmful or inappropriate practices by health workers that are never indicated (CIRCLE ALL THAT APPLY)				
Use of enema	A			
Public shaving	B			
Apply fundal pressure to hasten delivery of baby or placenta	C			
Lavage of uterus after delivery	D			
Slap newborn	E			
Hold newborn upside down	F			
Milking the newborn's chest	G			
Stretching of the perineum	H			
Shout, insult or threaten the woman during labor or after	I			
Slap, hit or pinch the woman during labor or after	J			
None of the above	Y			
Q604: Did you see any of the following practices done without an appropriate indication (CIRCLE ALL THAT APPLY)				
Manual exploration of the uterus after delivery	A			
Use of episiotomy	B			
Aspiration of newborn mouth and nose as soon as head is born	C			
Restrict food and fluids in labor	D			
None of the above	Y			
**REVIEW PARTOGRAPH AND/OR CHART FOR COMPLETENESS**				
**Question**	**Yes**	**No**	**DK**	**Go to**
Q605: Was there a newborn resuscitation? *(observer: check answer to Q500)*	1	0		No → Q611
EXAMINE CHART TO DETERMINE WHETHER THE HEALTH WORKER RECORDED THE FOLLOWING INFORMATION:				
Q606: Condition of the newborn at birth	1	0	8	
Q607: Procedures necessary to initiate breathing	1	0	8	
Q608: Time from birth to initiation of spontaneous breathing or time of death if unsuccessful	1	0	8	
Q609: Any clinical observations during resuscitation, including baby vital signs	1	0	8	
Q610: Final outcome of resuscitation measures	1	0	8	
EXAMINE PARTOGRAPH IF AVAILABLE				
Q611: was the partograph used to monitor labor?	1	0		No → Q630
Q612: Which partograph used	**Code**			
Old WHO partograph (latent phase)	1			
New WHO partograph (at 4cm dilatation)	2			

Other partograph	3			
**Question**	**Yes**	**No**	**DK**	**Go to**
Q613: Initiated use of partograph at the appropriate time according to partograph used (New WHO partograph starts at 4 cm; old version starts at 3 cm)	1	0	8	
EXAMINE PARTOGRAPH TO DETERMINE WHETHER THE HEALTH WORKER RECORDED THE FOLLOWING INFORMATION WHILE THE WOMAN WAS IN ACTIVE LABOR:				
Q614: Fetal heart rate plotted at least every half hour	1	0	8	
Q615: Cervical dilatation plotted at least every four hours	1	0	8	
Q616: Descent of head plotted at least every four hours	1	0	8	
Q617: Frequency and duration of contractions plotted at least every half hour	1	0	8	
Q618: Maternal pulse plotted at least every half hour	1	0	8	
Q619: BP recorded at least every four hours	1	0	8	
Q620: Temperature recorded at least every two hours	1	0	8	
Q621: OBSERVER: Did you see provider fill out partograph after delivery (with information that should be entered during labor)? (circle Don't Know if partograph use was not observed)	1	0	8	
EXAMINE PARTOGRAPH TO DETERMINE WHETHER THE HEALTH WORKER RECORDED THE FOLLOWING INFORMATION ABOUT THE DELIVERY				
Q622: Birth time	1	0	8	
Q623: Delivery method	1	0	8	
Q624: Birthweight	1	0	8	
**DATA EXTRACTION FROM PARTOGRAPH AND/OR CHART**				
Q625: Was action line on partograph reached?	1	0	8	No/DK → Q630
Q626: Record time action line was reached				
Q627: If action line reached on partograph, was any definitive action taken?	1	0	8	No/DK → Q630
Q628: Record time action was taken *(observer: enter 99:99 if unknown)*				
Q629: What definitive action was taken:	**Code**			
Consult with specialist	1			
Refer to other facility for specialist	2			
Prepare for assisted delivery	3			
Prepare for c-section	4			
Other (specify______________)	6			
FOR THE FOLLOWING QUESTIONS: EXAMINE PARTOGRAPH AND/OR CHART TO DETERMINE THE FOLLOWING INFORMATION. IF THE INFORMATION IS NOT IN THE CHART OR PARTOGRAPH, BUT THE OBSERVER KNOWS THE INFORMATION OR PREVIOUSLY RECORDED THE INFORMATION IN ANOTHER SECTION, HE OR SHE SHOULD FILL IN THEIR OWN ANSWER. IF THE INFORMATION IN THE CHART OR PARTOGRAPH DIFFER FROM OBSERVER'S INFORMATION, USE OBSERVER'S INFORMATION.				
Q630: Record age of woman				
Q631: Record the gravidity of the woman				
Q632: Record the parity of the woman prior to this delivery				
Q633: Time of admission to labor ward *(observer: enter 99:99 if unknown)*				
Q634: Centimeters dilated upon admission to labor ward *(observer: enter 99 if unknown)*				
Q635: Time membranes ruptured *(observer: enter 99:99 if unknown)*				
Q636: How did the membranes rupture?	**Code**			
Spontaneous	1			
Artificial	2			
Don't know	8			
Q637: Type of delivery				
Spontaneous vaginal	1			
Assisted (instrumented)	2			
Caesarean	3			
Don't know	8			
Q638: Time of birth *(observer: enter 99:99 if unknown)*				

Q639: Birthweight in grams *(observer: enter 9999 if unknown)*				
Q640: Record gestational age at birth in weeks *(observer: enter 99 if unknown)*				
**Question**	**Yes**	**No**	**DK**	**Go to**
Q641: Was she diagnosed with severe PE/E?	1	0	8	No→ Q643
Q642: Was baby delivered within 24 hours of PE diagnosis or within 12 hrs of eclampsia diagnosis?	1	0	8	
Q643: Did the mother have blood loss more than 500mL?	1	0	8	No→ Q645
Q644: Was she diagnosed with postpartum hemorrhage?	1	0	8	
Q645: Did the mother develop a fever of 38° C or higher during labor?	1	0	8	No→ Q647
Q646: Was she diagnosed with chorioamnionitis during labor?	1	0	8	
Q647: Were antibiotics administered to mother at any time?	1	0	8	No/DK → Q651
Q648: When were antibiotics administered? (CIRCLE ALL THAT APPLY)	**Code**			
1st stage	A			
2nd stage	B			
3rd stage	C			
Postpartum	D			
Q649: Why were antibiotics administered? (CIRCLE ALL THAT APPLY)				
Treatment for chorioamnionitis	A			
After prelabor rupture of membranes	B			
Preparation for C-section	C			
Routine/prophylactic	D			
Third stage/postpartum procedure	E			
Don't know	Z			
Q650: Which antibiotic was administered? (CIRCLE ALL THAT APPLY)				
Penicillin	A			
Ampicillin	B			
Gentamicin	C			
Metronidazole	D			
Cephalosporin	E			
Other	X			
Don't know	Z			
**Question**	**Yes**	**No**	**DK**	**Go to**
Q651: Is mother HIV positive? *(observer: circle Don't Know if status is unknown or was not discussed)*	1	0	8	No/DK → Q654
Q652: Was newborn given ARV(s)?	1	0	8	No/DK → Q654
Q653: Record type of ARV(s) given to newborn	**Code**			
NVP	1			
AZT	2			
3TC	3			
Don't know	8			

Q654: *PLEASE COMMENT ON THE QUALITY OF CARE PROVIDED:*
Was mother treated respectfully? Informed of procedures to herself and her baby? Was the situation chaotic or calm? Were there any major delays in needed treatment? If so, for what drugs/procedures and why? Were multiple health workers involved? Who? If maternal or newborn/fetal death occurred, describe the circumstances. Was the mother counseled about the death of newborn/fetus?

END OF SECTION 6
MAKE SURE THAT Q100, Q200, Q300, Q400, Q601-Q604 ARE ANSWERED BEFORE MOVING ON TO NEXT CLIENT

Section 7: Observation of Postpartum Hemorrhage

Question	Yes	No	Go to
Q700: Was this section observed?	1	0	No → Q800
CHECK THAT Q9 CLIENT CONSENT HAS BEEN FILLED IN. IF Q9 IS BLANK, COMPLETE COVER PAGE INCLUDING Q9 BEFORE STARTING PPH OBSERVATION			
Q701: Confirm that consent was received from client (or her proxy is she is incapacitated)	1	0	
Q702: Record time complication started			
RECORD WHETHER THE PROVIDER CARRIED OUT THE FOLLOWING STEPS AND/OR EXAMINATIONS: (SOME OF THE FOLLOWING STEPS MAY BE PERFORMED SIMULTANEOUSLY OR BY MORE THAN ONE PROVIDER). IF TIME OF EVENT IS UNKNOWN, ENTER 99:99. IF DOSE OF MEDICATION IS UNKNOWN, ENTER 99.			
**IMMEDIATE CARE**			
Q703: Monitors bleeding	1	0	No→Q705
Q704: How much bleeding was there (in mL)			
Q705: Performs uterine massage	1	0	No→Q707
Q706: Time massage performed			
Q707: Gives oxytocin	1	0	No→Q711
Q708: Record dose (in IU)			
Q709: Is route of administration intravenous (IV) through ringer's lactate or normal saline?	1	0	
Q710: Time oxytocin given			
Q711: Other uterotonic given	1	0	No→Q714
Q712: Which other uterotonic was given:	**Code**		
Ergometrine	1		
Syntometrine	2		
Misoprostol	3		
Q713: Time other uterotonic given			
**Question**	**Yes**	**No**	**Go to**
Q714: Performs abdominal exam for uterine contraction	1	0	No→Q716
Q715: Time exam performed			
Q716: Examines the vagina and perineum for lacerations and or cervical tear	1	0	No→Q718
Q717: Time exam performed			
Q718: Examines the placenta for completeness	1	0	No→Q720
Q719: Time exam performed			
Q720: Starts IV fluids	1	0	No→Q722
Q721: Time IV fluids started			
**FOLLOW UP CARE**			
Q722: Performs uterine exploration	1	0	No→Q724
Q723: Time procedure performed			
Q724: Performs uterine mechanical evacuation	1	0	No→Q726
Q725: Time procedure performed			
Q726: Performs manual removal of the placenta	1	0	No→Q728
Q727: Time procedure performed			
Q728: Performs bimanual compression of the uterus	1	0	No→Q730
Q729: Time procedure performed			
Q730: Performs aortic compression	1	0	No→Q732
Q731: Time procedure performed			
Q732: Uses balloon or condom tampanade	1	0	No→Q734

Q733: Time procedure performed			
Q734: Uses uterine sutures/cutgut	1	0	No→Q736
Q735: Time procedure performed			
Q736: Performs cardiac resuscitation	1	0	No→Q738
Q737: Time procedure performed			
Q738: Sends to surgery for hysterectomy	1	0	No→Q740
Q739: Time sent to surgery performed			
Q740: Performs blood clotting time	1	0	No→Q742
Q741: Time procedure performed			
Q742: Checks haemoglobin/haematocrit	1	0	No→Q744
Q743: Time procedure performed			
Q744: Requests blood grouping and crossmatching	1	0	No→Q746
Q745: Time procedure performed			
Q746: Gives blood products	1	0	No→Q749
Q747: Record number of units			
Q748: Time blood given			
Q749: Gives antibiotics	1	0	No→Q752
Q750: Which antibiotic was administered? (CIRCLE ALL THAT APPLY)	**Code**		
Penicillin	A		
Ampicillin	B		
Gentamicin	C		
Metronidazole	D		
Cephalosporin	E		
Other	X		
Don't Know	Z		
Q751: Time antibiotics given			
**Question**	**Yes**	**No**	**Go to**
Q752: Gives additional dose of oxytocin	1	0	No→Q756
Q753: Record dose (in IU)			
Q754: Is route of administration intravenous (IV)?	1	0	
Q755: Time oxytocin given			
Q756: Gives additional dose of other uterotonic	1	0	No→Q759
Q757: Which other uterotonic was given:	**Code**		
Ergometrine	1		
Syntometrine	2		
Misoprostol	3		
Q758: Time other uterotonic given			
**Question**	**Yes**	**No**	**Go to**
Q759: Is the woman's condition stable?	1	0	Yes→Q760
IF THE WOMAN'S CONDITION IS NOT STABLE, CONTINUE OBSERVING UNTIL SHE IS STABLE OR FOR AT LEAST 1 HOUR AFTER THE INITIAL EVENT (IF POSSIBLE). CHECK ANSWERS TO Q722-Q759 AS OBSERVATION CONTINUES.			
Q760: End time of observation			

(continued next page)

CASE DETAILS	
Q761: What is the woman's diagnosis (CIRCLE ALL THAT APPLY)	**Code**
Atonic uterus	A
Laceration	B
Incomplete expulsion of placenta	C
Placenta attached	D
Coagulopathy	E
Q762: At what stage of labor and delivery did the complication occur:	
At delivery	1
Postpartum (before discharge)	2
After discharge	3

Q763: *PLEASE WRITE A BRIEF SUMMARY OF THE CASE AND THEN DESCRIBE THE CONDITION OF THE WOMAN AT THE END OF OBSERVATION:*

Q764: *PLEASE COMMENT ON THE QUALITY OF CARE PROVIDED:*
Was the woman left alone at any point even if there was a danger of shock? Was she treated respectfully? Informed of procedures? Was the situation chaotic or calm? Were there any major delays in needed treatment? If so, for what drugs/procedures and why? Were multiple health workers involved? Who?

END OF SECTION 7 – RETURN TO APPROPRIATE SECTION TO CONTINUE OBSERVATION

Maternal and Newborn Quality of Care Survey

L&D Observation Checklist

Section 8: Observation of Severe Pre-eclampsia and Eclampsia			
**Question**	**Yes**	**No**	**Go to**
Q800: Was this section observed?	1	0	No → End section
CHECK THAT Q9 CLIENT CONSENT HAS BEEN FILLED IN. IF Q9 IS BLANK, COMPLETE COVER PAGE INCLUDING Q9 BEFORE STARTING PPH OBSERVATION			
Q801: Confirm that consent was received from client (or her proxy is she is incapacitated)	1	0	
Q802: Record the time complication started			
RECORD WHETHER THE PROVIDER CARRIED OUT THE FOLLOWING STEPS AND/OR EXAMINATIONS: (SOME OF THE FOLLOWING STEPS MAY BE PERFORMED SIMULTANEOUSLY OR BY MORE THAN ONE PROVIDER). IF TIME OF EVENT IS UNKNOWN, ENTER 99:99. IF DOSE OF MEDICATION IS UNKNOWN, ENTER 99.			
**IMMEDIATE CARE**			
Q803: Blood pressure taken	1	0	No→Q807
Q804: Record blood pressure: systolic			
Q805: Record blood pressure: diastolic			
Q806: Time blood pressure taken			
Q807: Urine checked for protein	1	0	No→Q810
Q808: Record result			
Q809: Time urine checked			
Q810: Were IV fluids started?	1	0	No→Q812
Q811: Time IV fluids given			
Q812: Magnesium sulfate given <u>intramuscular (IM)</u>?	1	0	No→Q815
Q813: Record dose in grams			
Q814: Time magnesium sulfate given IM			
Q815: Magnesium sulfate given <u>intravenously (IV)</u>?	1	0	No→Q818
Q816: Record dose in mL			
Q817: Time magnesium sulfate given IV			
Q818: Diazepam given	1	0	No→Q822
Q819: Record dose in mg			
Q820: Diazepam given intravenously (IV)?	1	0	
Q821: Time diazepam given			
Q822: Antihypertensive given	1	0	No→Q827
Q823: Which antihypertensive was given:	**Code**		
Hydralazine/ Apresoline	1		
Nifedipine	2		
Labetalol	3		
Methyldopa/ Aldomet	4		
Other (specify____________)	6		
Q824: Record dose in mg			
Q825: Route of administration			
Intramuscular (IM)	1		
Intravenous (IV)	2		
Oral/sublingual	3		
Q826: Time antihypertensive given			
**FOLLOW UP CARE**			
**Question**	**Yes**	**No**	**Go to**
Q827: Urinary catheter placed	1	0	No→Q829

Q828: Time catheter placed			
Q829: Labor induced or augmented (including artificial rupture of membranes)	1	0	No→Q831
Q830: Time labor induced or augmented			
Q831: Sent to surgery for c-section	1	0	No→Q833
Q832: Time sent for surgery			
Q833: Gives additional dose of magnesium sulfate	1	0	No→Q837
Q834: Record dose in grams			
Q835: Magnesium sulfate given intramuscular (IM)?	1	0	
Q836: Time magnesium sulfate given			
Q837: Gives additional dose of other medication	1	0	No→Q842
Q838: Which medication(s) was the client given *(*CIRCLE ALL THAT APPLY)	**Code**		
Diazepam	A		
Hydralazine/ Apresoline	B		→Q842
Nifedipine	C		→Q842
Labetalol	D		→Q842
Methyldopa/ Aldomet	E		→Q842
Other (specify____________)	X		→Q842
Q839: Record dose in mg for diazepam			
**Question**	**Yes**	**No**	**Go to**
Q840: Diazepam given intravenously (IV)?	1	0	
Q841: Time diazepam given			
Q842: Calcium gluconate given	1	0	No→Q844
Q843: Time calcium gluconate given			
**MONITORING**			
Q844: How many times in the first hour of observation was blood pressure taken	**Code**		
Blood pressure not taken during first hour	0		
Once	1		
Twice	2		
Three times	3		
Four times	4		
Five or more	5		
Q845: How many times in the first hour of observation were reflexes checked			
Reflexes not checked during first hour	0		
Once	1		
Twice	2		
Three times	3		
Four times	4		
Five or more	5		
Q846: How many times in the first hour of observation were respirations checked			
Respirations not checked during first hour	0		
Once	1		
Twice	2		
Three times	3		
Four times	4		
Five or more	5		
**Question**	**Yes**	**No**	**Go to**
Q847: Is the woman's condition stable?	1	0	
IF THE WOMAN'S CONDITION IS NOT STABLE, CONTINUE OBSERVING UNTIL SHE IS STABLE OR FOR AT LEAST 1 HOUR AFTER THE INITIAL EVENT (IF POSSIBLE). CHECK ANSWERS TO Q826-Q846 AS OBSERVATION CONTINUES.			
Q848: End time of observation			

CASE DETAILS			
Q849: What is the woman's diagnosis	**Code**		
Eclampsia	1		
Severe pre-eclampsia	2		
Other (specify____________)	3		
**Question**	**Yes**	**No**	**Go to**
Q850: Was the woman ever unconscious	1	0	
Q851: Did the woman experience convulsions	1	0	
Q852: At what stage of labor and delivery did the complication occur:	**Code**		
Before labor (including admissions from ANC)	1		
During labor	2		
At delivery	3		
Postpartum (before discharge)	4		
After discharge	5		

Q853: *PLEASE WRITE A BRIEF SUMMARY OF THE CASE AND THEN DESCRIBE THE CONDITION OF THE WOMAN AT THE END OF OBSERVATION*

Q854: *PLEASE COMMENT ON THE QUALITY OF CARE PROVIDED:*
Was the woman left alone at any point even if there was a danger of convulsions? Was she treated respectfully? Informed of procedures? Was the situation chaotic or calm? Were there any major delays in needed treatment? If so, for what drugs/procedures and why? Were multiple health workers involved? Who?

END OF SECTION 8

IF CLIENT IS IN LABOR OR HAS ALREADY DELIVERED, PLEASE RETURN TO APPROPRIATE SECTION TO CONTINUE OBSERVATION; IF CLIENT HAS NOT DELIVERED AND IS NOT IN LABOR PLEASE MAKE SURE THAT YOU HAVE ANSWERED Q100, Q200, Q300, Q400, Q500, Q601-Q604, AND Q700 BEFORE MOVING ON TO NEXT CLIENT.
